# A dc Method for the Absolute Determination of Conductivities of the Primary Standard KCl Solutions from 0 °C to 50 °C

**DOI:** 10.6028/jres.099.019

**Published:** 1994

**Authors:** Y. C. Wu, W. F. Koch, D. Feng, L. A. Holland, E. Juhasz, E. Arvay, A. Tomek

**Affiliations:** National Institute of Standards and Technology, Gaithersburg, MD 20899-0001; National Office of Measures, Budapest, Hungary

**Keywords:** absolute measurement, conductance, electrolytic conductivity, potassium chloride, primary standards, resistance standard

## Abstract

A new method for the absolute determination of electrolytic conductivity based on direct current and Potentiometric measurements is described. The unique design of the cell uses a removable center section whose length and cross-sectional area are accurately known. Two pairs of matched Ag, AgCl electrodes are used in a four terminal mode of resistance measurement. Measurements of the electrolytic conductivity of primary standard potassium chloride solution using [his novel dc conductance cell are compared with the currently adopted IUPAC and OIML recommendations. In addition, measurements have heen made of the electrolytic conductivity of a solution of potassium chloride having a molality of 1 mol/kg (mole KCl per kilogram H_2_O). The values so obtained over the temperature range of 0 °C to 50 °C are recommended as the new primary standards for electrolytic conductivity.

## 1. Introduction

The measurement of conductivity is based on the relation

κ≡1/ρ=G/R, (1)

where *κ* is the electrolytic conductivity (specific conductance) of an electrolyte solution; *ρ* is the resistivity; *G = l/A* is the cell constant of a cell having a length *l* between electrodes of area *A*; and *R* is the resistance. Of these three quantities, *G* is predetermined, and *R* is readily measurable, so that *κ* of an unknown solution can be determined. The determination of *G* is either by mechanical means (absolute measurement of the physical dimensions) or through an accurately and independently known *κ* (calibration measurement). When the primary standards for aqueous electrolytic conductivity are determined absolutely, the prime concern is accuracy; therefore an optimization of the three quantities is necessary. From Eq. (1) it is clear that when the *κ* to be determined is large, then either *G* needs to be large or *R*, the measured resistance will be small. However, there is a limitation on how large or how small either *G* or *R* can be, because the accuracy of both *G* and *R* depends upon the measuring instruments. Another practical restriction for *G* is space (i.e., the size of the controlled-temperature bath and the limit of the size of the connector) which limits the variation in *G* to a factor of about three (i.e., *l* greater than 30 cm is impractical). Two methods can be chosen for determining *R*: an ac bridge or a dc digital voltmeter (DVM). For solutions of low conductivity where *R* is high, the ac method is preferred because of lower experimental uncertainties. For solutions of high conductivity (low *R*), the dc method offers several advantages. Of these, the most obvious is the avoidance of the complications due to capacitance effects in the ac circuit. In addition, the lead resistance in a high conductivity (low resistance) ac measurement is a significant quantity, whereas for a four terminal dc measurement, the lead resistance is eliminated. The dc method was originated at the National Office of Measures in Hungary and was carried out at NIST.

The conductivities of a 1 demal^1^ (D) solution and a solution of potassium chloride having a molality^2^ of 1 mol/kg are relatively high (*κ* > 10 S/m) and, therefore, it is desirable to use the dc method to determine the relatively lower *R* (*R* about 100 Ω for *G* = 1000 m^−1^). The operating principle of the direct current method is based on Ohm’s law (*E*=*IR*). The potential difference of an *IR* drop can be measured accurately to 10^−7^ V to 10^−8^ V with a calibrated voltmeter. It is important that the current be kept relatively low to avoid heating the solution (*I*^2^*R*) and to minimize electrolysis (Faraday’s law). In addition, reversible electrodes are required to eliminate polarization effects. For KCl solutions, Ag, AgCl electrodes are suitable.

## 2. The Cell Design and the Assembly

**Fig. 1:**
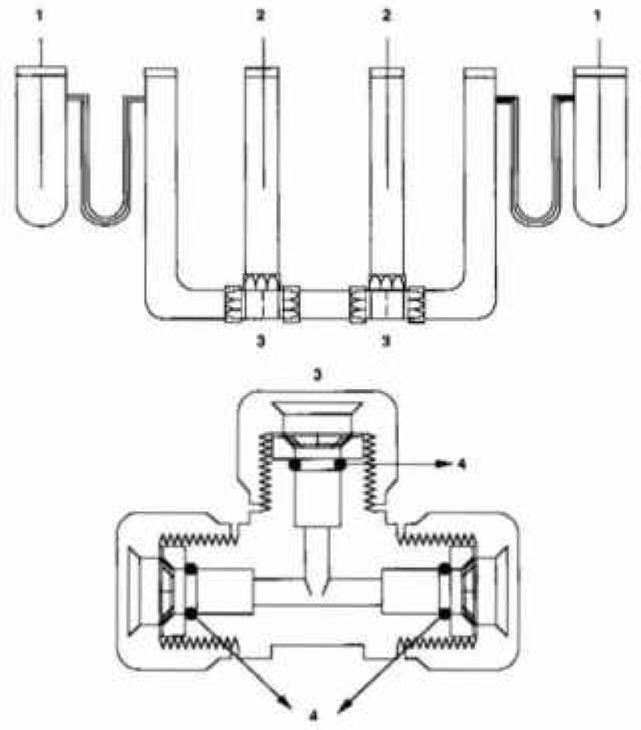
Schematic representation of the dc conductance Cell. 1—Pt or Ag-AgCl electrode 2—Ag-AgCl or calomel electrode (double junction) 3—nylon union tee 4—O-ring

For an absolute determination of conductivity the cell constant needs to be determined with basic standards, (e.g., length measurement). To accomplish this requirement, a precision bore pyrex tubing was cut into three pieces, in order that the center section of the cell could be removed and that the internal diameter and length of this center section could be measured accurately by physical methods. The two ends of the center section were polished to flatness, while the surfaces of the ends of the other two pieces which will join with the center section were ground to a roughness of about 25 μm. In this way, a slight gap was formed between the joined ends of the tubing to effect a miniscule flow of solution, when the sections are rejoined using a nylon union. The other two ends of the cut tubing are fused to pyrex tubing comprising the current compartments of the cell. A U-shaped capillary separates the current-electrode chamber from the connecting chamber to minimize the effect of electrolysis products on the measurement. A third tube is joined at each of the junctions of the center section with the end sections using a nylon-union tee. These compartments contain the potential leads for the measurements. The final assembly is shown schematically in Fig. 1.

The length, *l*, and internal diameter, *d*, of the center section were measured precisely at 25 °C by the Fabrication and Technology Division at NIST. Accordingly, at the 95 percent level of confidence, they are:

$$\begin{array}{l} l=(19.8500\pm 0.0002)\;\mathrm{cm}\\ d=(0.90010\pm 0.00008)\;\mathrm{cm}, \end{array}$$

whereupon,

$$\begin{array}{l} A=(0.63631\pm 0.00016)\;{\mathrm{cm}}^{2},\;\text{and}\\ G\equiv l/A=(31.195\pm 0.0053)\;{\mathrm{cm}}^{-1}. \end{array}$$

The temperature effect on the cell constant is similar to that reported previously [1], i.e.,

$$dG/dt=-\alpha_{g}\cdot G,$$ (2)

where *α*_g_, the thermal expansion coefficient of pyrex glass, is equal to 3.6 × 10^−6^/°C. Thus

G(t)=G(25°C)+(dG/dt)(t−25°C) (3)

or

$$G(t)=31.195\;{\mathrm{cm}}^{-1}-0.000112\;{\mathrm{cm}}^{-1}(t-25{}^{\circ}C).$$

The effect is less than 0.01% in the range of temperature of interest which is smaller than the uncertainty of the mechanical measurements. The value *G*(25°C) = 31.195 cm^−1^ is used throughout the experiments without corrections.

## 3. Experimental

### 3.1 Apparatus

**Fig. 2:**
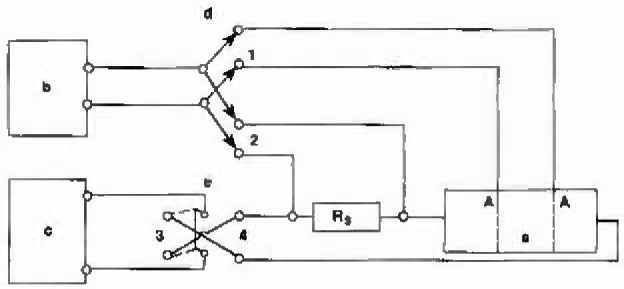
Schematic drawing of the operating and switching circuitry: a) The cell with gaps A,A, b) DVM, c) constant current supply. d) and c) double pole double throw switches; *R*,) standard resistor.

A high precision digital voltmeter (DVM), an accurate standard resistor, and a constant current supply are needed for the measurement of R. Both the DVM and the standard resistor have been calibrated by the Electricity Division at NIST. The constant current supply is capable of delivering current from 25 μA to 250 mA. Its stability is better than ±0.005% over 24 h. A schematic diagram of the operating circuit is shown in Fig. 2.

With switch “d” in the “2” position, and switch “e” in the “3” position, the current flows through the standard resistor and the conductance cell, and the *SR* drop across the standard resistor is measured by the DVM as *E*_*s*_ = *IR*_*s*_. *I* is thus determined. Moving switch “d” to the “1” position while switch “e” remains in the “3” position effects the measurement of the voltage between the potential electrodes in the conductance cell with current flowing, i.e., *E*_c_ = *IR*_c_. (The subscripts s and c refer to the standard resistor and the conductance cell, respectively.) Switching “e” to the “4” position reverses the flow of current and the polarity of the current electrodes. If there is any disparity between the potential electrodes in the conductance cell, it will manifest itself in the voltage measurement by reversing the current. Therefore, the constancy of the current is established. Hence, a data point at a single current consisted of four measurements as shown in Table 1.

Two pairs of matched Ag, AgCl electrodes of the type used in the emf/pH research effort at NIST were used in the conductance cell, one pair for the current electrodes, the other for the potential electrodes. The preparation and calibration of these electrodes have been described in conjunction with the pH studies [1]. All four electrodes were within ±0.05 mV of each other. Ag, AgCl electrodes were used instead of Pt electrodes, even for the current electrodes, to minimize or eliminate the effects of electrolysis and polarization.

Three types of constant temperature baths were used in this study; oil, water, and ice. The oil bath has been described elsewhere [2]. An old water bath was modified to make more room for the conductivity cell. Its temperature was controlled to ±0.001 °C to ± 0.002 °C for temperatures above 0 °C. At 0 °C, an ice bath was used.

### 3.2 Materials

SRM 999 KCl was used to prepare the solutions. It was ignited at 500 °C for 4 h and stored in a desiccator before use. Distilled and deionized water with conductivity <0.2 μS/cm was employed. Solutions were prepared by weight and buoyancy corrections were made.

### 3.3 Procedure

The glassware of all sections of the cell was cleaned with chromic acid, then, HCl, rinsed with water, and soaked in water overnight. After the sections were dried, the cell was assembled. Solution was added and the electrodes were placed in the appropriate chambers. The whole cell assembly was put into the bath, and its temperature was adjusted and controlled at the designated point. Usually, the starting temperature was 25.000 °C, and a steady state was reached after about 30 min. The wiring to the electrodes was connected according to the scheme shown in Fig. 2, and the measuring sequence commenced. It took approximately 3 s for a reading. There were four readings for a selected current, two each for each direction (*E*_s_ and *E*_c_) as described above. Each data point was taken as the mean of three different currents. At each temperature, the current flowed through the cell for approximately 30 s. Thus, the power dissipated in the cell was less than 1 mW, and its effect was much smaller than a millidegree per second change for the solution. Therefore, there was no significant heating effect.

### 3.4 Results

According to Eq. (1), the determination of the conductivity of the solution requires the measurement of resistance, *R*. For the dc method, *R* is determined by Ohm’s law as *E/I*. The current, *I*, is determined by means of a standard resistor, *R*_s_, where *E*, the potential difference (or voltage drop) is read from a digital voltmeter, DVM. The results for *R* and the electrolytic conductivity of a 1 D solution and a solution of potassium chloride having a molality of 1.0 mol/kg at various temperatures are listed in Tables 2 and 3; the temperature coefficients are also given.

## 4. Discussion

The values shown in Tables 2 and 3 are the results of three series of measurements. In each series the cell was disassembled, cleaned, dried and reassembled. In each of the processes, the gaps between the ends of the center tube and the end tubes could be affected. This limits the reproducibility to ±0.01%.

Additional sources of uncertainty are derived from (1) the cell constant, (2) the solution, and (3) the electrical measurement. These sources are examined as follows:

### 4.1 The Cell Constant

There are three limitations that restrict the improvement of accuracy, viz., the size of the precision bore tubing, the union-tee fitting that holds the tubing together, and the accuracy of the measuring instrument. For the latter, the uncertainty (2σ estimate) of the dimensional measurement at the Fabrication and Tehnology Division at NIST is 1 μm. Therefore, a larger bore and longer tubing would be needed to reduce the relative uncertainty. However, at present, the largest commercially available nylon union-tee limits the outside diameter of the tubing to 1.25 cm. This alone limits the reduction of the relative uncertainty for the cell constant. In this study, the cell constant from 11 measurements was (31.195 ±0.0053) cm^−1^, at the 95% level of confidence.

### 4.2 The 1 D KCl Solution and KCl Solution Having a Molality of 1.0 mol/kg

The KCl and water have been proved to be adequately pure [2]. The relative expanded uncertainty (2*σ* estimate) associated with the weighing was 0.002%; the balances were calibrated and checked with NIST weights. Another source of uncertainty was the evaporation of solution in the transferring process and in rotating the cell to get rid of the trapped air bubble(s). The relative expanded uncertainty (2*σ* estimate) associated with the preparation of the solution is estimated to be 0.003%.

### 4.3 Electrical Measurements

In this category, there should only be negligible uncertainties (2*σ* estimate) from systematic effects (0.001%), because the constant current source is very stable (approximately 0.0005%), and the DVM (uncertainty specified by the manufacturer 0.0005%) and the standard resistor have been calibrated with primary standards, as discussed above.

To sum up: the relative expanded uncertainty at the 95% level of confidence is estimated to be *U* = 0.018%.

## 5. Conclusion

The electrolytic conductivity values for the 1 D solution and the solution of potassium chloride having a molality of 1.0 mol/kg have been determined absolutely from 0 °C to 50 °C in intervals of 5 °C by the dc method.

These values are listed in Table 4, together with the 1 D values recommended by the Organisation Internationale de Metrologie Légale (OIML) [4] and converted to ITS-90 [3]. They agree to ±0.02%, which is within the experimental uncertainty.

Because of the improvement in accuracy, the enhancement of the temperature range from 0 °C to 50 °C, and the inclusion of the molality scale, the values listed in Table 4 are recommended as the primary electrolytic conductivity standards.

## Figures and Tables

**Table 1:** Measurement Sequence

Measurement No.	Switch “d”	Switch “e”
1	2	3
2	1	3
3	1	4
4	2	4

**Table 2:** Electrolytic conductivity values for a solution of potassium chloride having a molality of 1.0 mol/kg at temperatures from 0 °C to 50 °C

*t*/°C	*R*/Ω^a^	*κ*/(S/cm)		
		Observed	Smoothed^b^	Dev. %^c^
0	491.32	0.063 493	0.063 487	0.009
5	433.17	0.072 016	0.072 030	−0.020
10	385.96	0.080 825	0.080 844	−0.023
15	347.06	0.089 884	0.089 900	−0.018
20	314.54	0.099 175	0.099 170	0.005
25	287.14	0.108 64	0.108 62	0.019
30	263.83	0.118 24	0.118 24	0.000
35	243.74	0.127 99	0.127 97	0.014
40	226.40	0.137 79	0.137 81	−0.017
45	211.16	0.147 73	0.147 72	0.005
50	197.82	0.157 69	0.157 67	0.015

a Each *R* value is the mean of six values measured at different currents with a standard deviation of the mean no greater than 0.035%.

b Equation: *κ*/(S/cm)=0.063 488 2 + 1.67913 × 10^−3^ *t* + 6.007 81 × 10^−6^ *t*^2^ −3.837 02 × 10^−8^ *t*^3^.

c Dev. % = 100 (*κ*−*κ*_Smoothed_)/*κ*_Smoothed_.

**Table 3:** Electrolytic conductivity values for 1 D KCl solution at temperatures from 0 °C to 50 °C

*t*/°C	*R*/Ω^a^	κ/(S/cm)		
		Observed	Smoothed^b^	Dev. %^c^
0	478.95	0.065 132	0.065 135	−0.005
5	422.44	0.073 845	0.073 860	−0.021
10	376.43	0.082 870	0.082 871	−0.002
15	338.56	0.092 140	0.092 136	0.005
18	318.95	0.097 805	0.097 804	0.001
20	306.97	0.101 62	0.101 62	0.001
25	280.28	0.111 30	0.111 30	−0.002
30	257.58	0.121 11	0.121 11	−0.002
35	238.10	0.131 02	0.131 05	−0.026
40	221.18	0.14 104	0.141 08	−0.031
45	206.43	0.151 12	0.151 15	−0.022
50	193.40	0.161 29	0.161 24	0.033

a See footnote “a” to Table 2.

b Equation: *κ*/(S/cm) = 0.065 135 + 1.714 × 10^−3^ *t* +6.414 l × 10^−6^ *t*^2^ −4.502 8 × 10^−8^ *t*^3^.

c See footnote “c” to Table 2.

**Table 4:** Recommended electrolytic conductivity values for solutions of potassium chloride having a molality or 1.0 mol/kg, and for 1 D solutions

*t*/°C	KCl solution 1.0 mol/kg	*κ*/(S/cm)	
		KCl solution 1 D (present work)	KCl solution 1 D (OIML(4))
0	0.063 487	0.065 135	0.065 144
5	8.072 030	0.073 860	
10	0.080 844	0.082 871	
15	0.089 900	0.092 136	
18		0.097 804	0.097 82
20	0.099 170	0.101 62	
25	0.108 62	0.111 30	0.111 32
30	0.118 24	0.121 11	
35	0.127 97	0.131 05	
40	0.137 81	0.141 08	
45	0.147 72	0.151 15	
50	0.157 67	0.161 24	

## Footnotes

1 The demal (D) is a designation unique to electrolytic conductivity and is defined at only three points: a 1 D solution, as adopted by OlML, is defined as 71.1352 g KCl per kg solution; a 0.1 D solution is defined as 7.4l913 g KCl per kg solution; and a 0.01 D solution is defined as 0.745263 g KCl per kg solution.

2 Molality is a designation in common usage in physical chemistry and thermodynamics. The molality of solute B is the amount of substance of solute B in a solution divided by the mass of the solvent. A solution of potassium chloride having a molality of 1 mol/kg is prepared by dissolving 1 mole of KCl in 1 kg of H_2_O.
